# Enzyme and Antioxidant Activities of Malted Bambara Groundnut as Affected by Steeping and Sprouting Times

**DOI:** 10.3390/foods11060783

**Published:** 2022-03-08

**Authors:** Adeola Helen Adetokunboh, Anthony O. Obilana, Victoria A. Jideani

**Affiliations:** Department of Food Science and Technology, Cape Peninsula University of Technology, Bellville 7535, South Africa; helinaa2002@gmail.com (A.H.A.); obilanaa@cput.ac.za (A.O.O.)

**Keywords:** Bambara groundnut, α-amylase, β-amylase, total polyphenols, antioxidant, steeping, sprouting

## Abstract

Bambara groundnut (BGN) is termed a complete food due to its nutritional composition and has been researched often for its nutritional constituents. Malting BGN seeds have shown improved nutritional and functional characteristics, which can be used to produce an amylase-rich product as a functional ingredient for food and beverage production in homes and industries. The aim of this study was to investigate the enzyme and antioxidant activities of malted BGN affected by steeping and sprouting times. BGN was malted by steeping in distilled water at 25–30 °C for 36 and 48 h and then sprouted for 144 h at 30 °C. Samples were drawn every 24 h for drying to study the effect of steeping and sprouting times on the moisture, sprout length, pH, colour, protein content, amylase, total polyphenols, and antioxidant activities of the BGN seeds. The steeping and sprouting times significantly affected the BGN malt colour quality and pH. The protein content of the malted BGN seeds was not significantly different based on steeping and sprouting times. Steeping and sprouting times significantly affected the α- and β-amylase activities of the BGN seeds. The activity of amylases for 36 and 48 h steeping times were 0.16 and 0.15 CU/g for α-amylase and were 0.22 and 0.23 BU/g for β-amylase, respectively. Amylase-rich BGN malt was produced by steeping for 36 h and sprouting for 96 h. Amylase-rich BGN malt can be useful as a functional food ingredient in food and beverage formulations.

## 1. Introduction

Malting is the incomplete germination of cereal grains and pulses under controlled water, temperature, and humidity [1,2,3,4,5]. It involves three stages: steeping, sprouting, and drying (kilning), carefully monitored because every process stage affects the end product [4,6]. The main objective of malting is to encourage hydrolytic enzyme development because un-germinated cereals and legumes cannot develop enzymes [5,7]. Sprouting and acrospires form during the malting process, and enzymes become active, altering the grain structure, and resulting in a malt product used in the brewing, food, and beverage industries. The sequence of substantial changes in metabolites and enzyme activities in the resultant malts, on the other hand, is time-dependent [4,8,9]. The seed embryo releases gibberellic acid (GA), which moves to the aleurone to induce hydrolytic enzymes [10]. The enzymes released catalyse the breakdown of cell wall reserves (polysaccharides and starchy energy) necessary for sprouting and malt production [10,11,12].

The malting process metabolically makes the protein soluble, increases the enzyme activity, breaks down starch into simple sugars, and develops colour and flavour [4,10,13]. The essential enzymes for the diastatic power measurements are the α- and β-amylases [4,14]. Apart from the α- and β-amylases that hydrolyse starch, there are many metabolic changes and modifications during sprouting and malting [1,10,15]. Sprouting seeds in plant science signifies a vital stage depending on time [16,17]. The steeping, sprouting, and drying times affect the changes and modifications in the malted cereals and legumes [18,19,20,21,22,23,24].

Sprouting is an effective and inexpensive technology used to improve (modification and increase nutritional components) cereal and legume grain quality [25,26,27,28]. Sprouting has been established to improve the nutritional properties, to increase the essential nutrients, to lower the anti-nutrients, and to increase the antioxidant content [27,29,30,31,32,33]. There are many physical, chemical, and biological changes inside the seed during the sprouting stage [28]. For example, the activation of the hydrolytic enzymes, hydrolysing proteins, polysaccharides, and fats leads to increased nutritional and antioxidant contents [34,35].The biological changes in sprouted grains lead to ease of processing [32,36,37] and the creation of colour, odour, flavour, and functional properties [26,27,32,33,35,36,37,38,39,40,41]. In addition, it has been established through studies that eating germinated cereals and legumes may help in the reduction of chronic diseases such as cancer, diabetes, hypertension, hyperlipidaemia, obesity, and heart diseases [26,27,31,35].

Native to north-east China (Manchuria), soybean (Glycine max) malting characteristics has been researched, especially on soaking, sprouting, and drying durations [42,43,44,45]. Soybean’s malting process improved its nutritional content and removed anti-nutrients [46]. In addition, some functional, active components present in low content in un-germinated soybean were increased by germination [46,47]. Such components include soy isoflavones, γ-aminobutyric acid (GABA), polyphenols, and antioxidants [48,49,50,51,52,53,54]. These components change the nutritional, physical, functional, and health benefits of malted soybeans, which has contributed to the development of healthy soybean food products [55,56,57].

Bambara groundnut (BGN), a legume indigenous to Africa, has also been researched due to its popularity such as soybean. Bambara groundnut is known for its various nutritional and therapeutic values [58,59,60]. Research has proven BGN to be resilient and able to withstand drought conditions compared with other legumes, with the ability to produce high yields [61,62,63,64,65,66]. New and improved products have been developed from malted and un-malted (raw BGN) to encourage its use. Some of the new products from BGN through research include yoghurt, milk (powdered and liquid), and value-added snacks [67,68,69,70].

Bambara groundnut has also been malted to study its chemical properties and functional use in new food formulation and its therapeutic potentials. It was malted to investigate the effects on the milling performance and acceptability of the malted seeds for the production of okpa [71]. Akpapunam et al. [72] investigated the malting time effect on BGN flour chemical composition and its functional properties. The profiling of the phenolic compounds in sprouted BGN by [73] showed an increase by 1.3-fold total polyphenol content after sprouting, revealing new emerging compounds. Additionally, sprouted BGN flours caused a decline in phytic acid, tannin, and trypsin inhibitor, which resulted in the improvement of protein content, insoluble dietary with the enhancement of the trace minerals, amino acids, digestible starch, and in vitro protein digestibility [74]. Sprouting has been shown to be effective in the decrease of anti-nutritional components, improving the nutritional and functional properties of BGN [75,76]. However, there is a need to know the characteristic physicochemical changes that occur during the malting process of BGN seeds and its use as a functional ingredient for food and beverage production in homes and industries. Therefore, this chapter’s objective was to establish the physicochemical characteristics, enzymes, total polyphenolics, and antioxidant activities of malted BGN seeds affected by steeping and sprouting times.

## 2. Materials and Methods

### 2.1. Source of Materials, Reagents, and Equipment

The BGN seeds were purchased from Triotrade Johannesburg, South Africa, and used as received without sorting into the colours. The chemicals and reagents were of analytical standards. α- and β-amylase kits were from Megazyme Ltd., Ireland. All other equipment was from the Department of Food Science and Technology and Oxidative Stress Research Centre, Cape Peninsula University of Technology, Cape Town, South Africa.

The equipment and instruments used in this study were the ten trays of hot air Excalibur Food Dehydrator (Excalibur, Sacramento, CA, USA), LECO CN 628 Dumas nitrogen analyser (Leco Corp., St. Joseph, MI, USA), the centrifuge (Avanti^®^ J-E centrifuge JSE111330, Beckman coulter Inc., Indianapolis, IN, USA), and Thermos Scientific Multiskan plate reader spectrophotometer (Thermo Scientific, Waltham, MA, USA). Others are the pH meter (Hannah checker pH meter, Model HI1270), a water bath, and Colour Flex EZ (Model TC-P III-A, Tokyo Denshoku Co., Ltd., Tokyo, Japan). The sample treatments and analyses carried out in this section consisted of the sprout length, moisture, colour, pH, protein content, α- and β-amylase activities (Ceralpha & β-amylase enzymatic kit), total polyphenols (Folin–Ciocâlteu Reagent Assay (FCR) method), and antioxidant activities (Ferric Reducing Antioxidant Power Assay (FRAP) and 2,2-diphenyl-1-picrylhydrazyl assay (DPPH) methods).

### 2.2. Malted Bambara Groundnut Production Process

The raw BGN were spread out in trays to facilitate the removal of foreign materials and broken and poorly developed seeds. Distilled water was used to wash the grains to adequately remove dirt and dust particles. The cleaned BGN seeds were divided into two equal portions of 1400 g each and were steeped at 36 and 48 h due to their hard nature [77,78]. The two batches of cleaned BGN seeds (1400 g) were steeped at 25 °C for 12 h, followed by a 12 h air rest (25 °C) and a second steep (12 h, 25 °C) for 36 h steeped seeds. While the 48 h steeped BGN seeds were steeped at 25 °C for 24 h, followed by a 12 h air-rest (25 °C) and a second steep (12 h, 25 °C). The steeped grains were air-rested 12 hourly by spreading them on 45 by 30 cm plastic tray baskets at 25 ± 30 °C to allow air into the grains. Steeping was performed with 7 L of distilled water using two 25 L round white buckets for 38 and 48 h at 25 °C until they absorbed between 41 and 43% of their initial weight in water to initiate sprouting. The 36 and 48 h steeped BGN seeds increased in weight to 2394 and 2702 g, respectively and were divided into six equal portions. The seeds were spread out on the plastic tray baskets, arranged inside the side-by-side Macadam proofer (2250 by 1000 mm in size) at 99% humidity and 30 °C from 24 to 144 h.

Distilled water (10 mL) was sprinkled on the sprouting seeds every 12 h. Ten samples of the sprout length in triplicate from the sprouted seeds for each batch every 24 h were measured using the Vernier calliper. Samples were drawn at 24, 48, 72, 96, 120, and 144 h of germination time and dried in a hot air Excalibur Food Dehydrator (Excalibur, Sacramento, CA, USA) maintained at 55 °C for 24 h. The dried samples were milled using Waring Laboratory Science blender model 7009G (Waring Laboratory Science, CT, USA), then packaged in zip lock bags, and stored at −18 °C until further analysis. The samples were analysed for the physicochemical characteristics (pH, colour, sprout length, and moisture), amylase, total polyphenolic content, and antioxidant activities.

### 2.3. Physicochemical Analysis of Bambara Groundnut Malt

#### 2.3.1. Sprout Length and Moisture Uptake of the Bambara Groundnut Green Malts

The length of the BGN green malt sprouts was measured with the Vernier calliper by measuring ten seeds in triplicate from each day of sprouting following the method of [79]. The results (in centimetres) were the average values from a triplicate set of ten seeds of the malted BGN seeds. According to [80], the moisture uptake percentage was determined by measuring the 36 and 48 h BGN seeds on the electronic laboratory balance before steeping. After steeping, the seeds were strained and blotted with a towel to remove the excess water on the surface before weighing. Finally, the moisture uptake percentage (wet basis) was calculated according to Equation (1).

(1) $$\frac{W_{2-}W_{1}}{W_{1}}\times 100$$

W_1_ is the weight before steeping, and W_2_ is that after steeping.

#### 2.3.2. pH Determination of Bambara Groundnut Malts

Following the method of [81], a slurry with 10 g milled BGN malt and 40 mL distilled water was prepared in 50 mL centrifuge tubes. The vortex mixer was used to mix the BGN malt and distilled water thoroughly. The mixtures were kept at room temperature for 1 h and centrifuged at 1500× *g* for 10 min. The decanted liquid pH was measured in triplicate using a laboratory pH meter (Hannah checker pH meter, Model HI1270), standardized with buffers 4 and 7.

#### 2.3.3. Colour Determination of Bambara Groundnut Malts

The samples’ colour measurements were analysed using Colour Flex EZ (Hunter Lab, Reston, VA, USA), 25 mm aperture set for daylight illumination D65, and 10° standard observer angle following the method of [82]. The instrument’s calibration was performed using standard black (L* = 8.47, a* = −0.96, b* = 2.79) and white (L* = 93.41, a* = −1.18, b* = 0.75) tiles.

The colour coordinate measurement was in triplicates by measuring 5 g of the samples into a glass sample cup (Hunter Lab 04720900, 6.4 cm) with an internal diameter of 6.4 cm following the method by [83]. Measurement was conducted using the Commission Internationale de l’Eclairage’s (CIE) L*a*b*, where L* (0 = black and 100 = white), a* (−a* = greenness, and +a* = redness), and b* (−b* = blueness and +b* = yellowness). The chroma and hue values were calculated using the method of [34], as shown in Equations (2) and (3).

(2) $$C=\sqrt{{a*}^{2}+{b*}^{2}}$$

where C = chroma; a* = redness; and b* = yellowness:

(3) $$h^{{}^{\circ}}=\tan^{-1}\left(\frac{b^{*}}{a^{*}}\right)$$

where h° = hue angle, a* = redness, and b* = yellowness.

#### 2.3.4. Bambara Groundnut Malts Protein Content Determination

The LECO CN 628 Dumas nitrogen analyser (Leco Corp., St Joseph, MI, USA) was used to determine the nitrogen content of the samples according to the method of the Association of Official Analytical Chemists [84]. Five blanks, EDTA standard, and ProNutro control sample were first analysed and then sampled (0.09 mg) in duplicate, wrapped and tightly folded in tin foil cups P/N: 502-186-200.

Combustion of the samples was carried out in pure oxygen at a temperature of 950 °C in the reactor consisting of the combustion catalyst, where a gaseous mix containing carbon dioxide, water, and nitrogen formed (CO_2_, H_2_O, NO, and NO_2_). The designated columns then absorbed the gases, removed oxygen, and converted nitrogen oxides into nitrogen. The residual CO_2_ and H_2_O were extracted by passing through a thermal conductivity column carried by helium gas. The Dumas Nitrogen analyser measured the nitrogen content. The crude protein was calculated by multiplying the measured nitrogen by the protein factor of 6.25 expressed in percentage following [85,86].

### 2.4. Determination of α- and β-Amylase Activities of BGN Malts

The method of Montanuci et al. [86] for α- and β-amylases content determination during the malting process was followed. The α- and β-amylase enzymes were determined through the enzymatic Ceralpha kit (K-CERA, Megazyme Southern Cross Rd, Bray, Co. Wicklow, A98 YV29, Ireland) and the enzymatic kit β-amylase (Megazyme, K-BETA3) as detailed in Section 2.4.1 and Section 2.4.2, respectively. All analyses for enzymatic activity were performed in triplicate.

#### 2.4.1. Alpha-Amylase Assay Procedure (Ceralpha Method)

The milled 36 and 48 h steeped BGN malts (3 g) were weighed separately into 50 mL conical flasks. To each flask, 20 mL of extraction buffer solution of pH 5.4 was added and the contents of the flask were stirred vigorously using the vortex mixer. The samples were then extracted for 20 min at 40 °C in the incubator with occasional stirring using a vortex mixer. After extraction, 25 mL of each sample was measured into 50 mL centrifuge tubes and centrifuged using the Centrifuge 5810R at 1000× *g* for 10 min. Finally, the sample extracts were decanted into 25 mL centrifuge tubes for the assay procedure.

The assay was carried out by measuring 0.2 mL aliquots of Megazyme un-buffered amylase HR reagent into 25 mL centrifuge test tubes. It contains blocked p-nitrophenyl maltoheptaoside (BPNPG7, 54.5 mg) and thermostable α-glucosidase (125 U at pH 6.0). The two were pre-incubated at 40 °C for 5 min. The 0.2 mL sample extracts were also pre-incubated at 40 °C for 5 min and added directly to the tubes’ containing the 0.2 mL of the amylase HR reagent solution. These were incubated at 40 °C for 20 min, and precisely 3.0 mL of stopping reagent containing 10 g of tri-sodium phosphate in 1 L of distilled water pH adjusted to 11.0 was added. The contents of the tube were vigorously stirred using the vortex mixer. The absorbance of the solutions was read in triplicate using the Thermo Electron Corporation Multiskan Spectrum set at 400 nm against distilled water.

#### 2.4.2. β-Amylase Assay Procedure (Betamyl-3 Method)

The milled 36 and 48 h steeped BGN malts of 0.5 g were weighed into 25 mL centrifuge tubes. Five millilitres of the Megazyme extraction buffer (Tris/HCl 25 mL, 1 M, pH 8.0 plus disodium EDTA of 20 mM and sodium azide of 0.02% *w/v* diluted in distilled water) were added to the sample tubes. The enzymes were extracted for 1 h at room temperature, with repeated stirring on the vortex mixer. The mixtures were centrifuged using the Eppendorf Centrifuge 5810/5810 R at 2000× *g* for 10 min. Immediately after centrifugation, 0.2 mL of the filtrate were added to 4.0 mL of the dilution buffer containing MES dilution buffer 48 mL, 1 M, pH 6.2 plus disodium EDTA 20 mM, BSA 10 mg/mL, and sodium azide of 0.09% *w/v*. This mixture was then used for the assay of β-amylase activities.

The assay of the β-amylase was carried out by dispensing an aliquot of 0.2 mL of the diluted BGN malt samples into the 25 mL centrifuge tubes. The tubes were pre-incubated at 40 °C for 5 min. After incubation, 0.2 mL of pre-incubated Megazyme Betamyl-3 substrate solution containing p-nitrophenyl-β-D-maltotrioside (PNPβ-G3) plus β-glucosidase (50 U) and stabilisers were added to each diluted sample and stirred on the vortex mixer. These mixtures were incubated at 40 °C for 10 min. After that, 3.0 mL of the stopping reagent (10 g of Tris buffer (Megazyme cat. no. B-TRIS500) in 900 mL of distilled water, pH adjusted to 8.5) was added. The contents were mixed using a vortex mixer. The absorbance of the solutions was read at 400 nm against distilled water using a Thermo Scientific Multiskan microplate spectrophotometer.

### 2.5. Determination of Total Polyphenols and Antioxidants Activities of Bambara Groundnut Malts

The Folin–Ciocâlteu reagent (FCR), ferric reducing antioxidant power (FRAP), and 2,2-diphenyl-1-picrylhydrazyl (DPPH) assay methods used for the determination of polyphenolic and antioxidants activities were followed [87,88,89] as detailed in Section 2.5.1 to Section 2.5.3.

#### 2.5.1. Total Polyphenols Activity Determination by Folin–Ciocâlteu Reagent Assay (FCR) Method

The analysis used the Folin–Ciocâlteu reagent with Gallic acid as the standard to quantify total polyphenols in BGN malts. The phenolic contents were determined by weighing 500 mg of each sample into screw-cap tubes. The BGN malt samples extraction was carried out with 10 mL of 70% methanol mixed with 0.1% HCL using a vortex mixer. The mixtures were then centrifuged using the Eppendorf Centrifuge 5810/5810 R at 4000× *g*, 21 °C for 5 min. The supernatant was analysed using the Folin–Ciocâlteu assay. Twenty-five microlitres of the sample’s supernatant were mixed with 125 µL of 0.2 M Folin–Ciocâlteu reagent and 100 µL of 7.5% Na_2_CO_3_ solution in a 96-well clear plate. The absorbance was read with a Thermos Scientific Multiskan microplate spectrophotometer reader (734 nm at 25 °C) after a 2 h incubation period. The Gallic acid constructed the standard calibration curve. The results were expressed as milligram Gallic acid equivalents (GAE/g).

#### 2.5.2. Antioxidant Activities Determination by Ferric Reducing Antioxidant Power Assay (FRAP) Method

Five hundred milligrams of the 36 and 48 h steeped BGN malts were weighed into 50 mL screw-cap tubes. Ten millilitres of 70% methanol (containing 0.1% HCl) were added to the samples of the screw-cap tubes. The samples were mixed with a vortex then centrifuged at 4000 rpm for 5 min. The supernatants (10 µL each) were pipetted into microplate wells in triplicates. Three hundred microliters of the FRAP reagent were added to each sample in the microplate wells. The samples were incubated for 30 min at 37 °C, and absorbance was read at 593 nm using the Thermo Scientific Multiskan microplate spectrophotometer. The results were expressed as milligram ascorbic acid equivalents (AAE/g).

#### 2.5.3. Antioxidant Activities Determination by 2,2-Diphenyl-1-Picrylhydrazyl Assay (DPPH) Method

The 36 and 48 h steeped BGN malt free radical scavenging ability was determined using the DPPH radical (25 mg/L) in 70% methanol. Each of the samples was mixed with 0.275 mL DPPH solutions. The samples and standards were incubated at 37 °C for 30 min in the dark, and absorbance was read at 517 nm using the Thermos Scientific Multiskan microplate spectrophotometer. The standard was Trolox, and the results were expressed as micromole Trolox per gram.

### 2.6. Data Analysis

IBM Statistical Package (IBM SPSS, version 26, 2018) was used for data analysis. All data were collected in triplicate, and the results were expressed as the mean ± standard deviation. The results were subjected to multivariate analysis of variance (MANOVA) when normality was not violated and the Kruskal–Wallis H test when normality is violated to determine the mean differences between treatments. Duncan’s multiple range tests were conducted to separate the means where differences existed at *p* ≤ 0.05 (IBM SPSS version 26).

## 3. Results and Discussion

### 3.1. Water Absorption of Steeped Bambara Groundnut Seeds

There was a 41.5% (SD = 5.72) increase in water uptake after the BGN seeds’ steeping for 36 h and a 48.2% (SD = 5.72) increase for the 48 h steeping. The increase showed a difference of 6.66% in water uptake of the two steeping regimes, indicating that the steeping time affected the water uptake of the seeds, as stated by [79,90,91]. Legume seeds such as BGN have hard seed coats that make them impermeable to water [90]. The slow water uptake of legumes has been attributed to their hilum [91]. The hilum is a scar on the legume seeds that marks the attachment point to the seed, where water enters the seed coat and cotyledon [92]. Once the seed coat is fully hydrated, it allows for water uptake by diffusion until the equilibrium moisture content is achieved [91,93,94].

### 3.2. Effect of Steeping and Sprouting Times on the Sprout Length of Bambara Groundnut Green Malts

The sprout length increased by 2.33 cm for the 36 h steeped seeds and by 1.99 cm for the 48 h steeped seeds. The steeping times significantly (*p* = 0.004) affected the sprout development where 48 h steeped BGN seeds were shorter than the 36 h steeped seeds. The longer steeping regime slowed sprout growth and thus the reduction in sprout length in the 48 h steeped seeds. The longer steeping duration resulting in reduced sprout length was reported by [79]. The shortest sprout length was observed in Thai rice malt cultivars (*Oryza sativa* L. Indica) steeped for 72 h, followed by 48 and 24 h. Additionally, the authors of [95] noted that steeping at 48 h had longer sprouts than 72 h steeped rice grain. However, the authors of [96,97,98,99] noted that longer steeping encourages an increase in sprout length, resulting in higher water absorption in Korean red bean, Mung bean, buckwheat, and millet, respectively. The increase in sprout length was attributed to steeping, which encourages respiration and energy metabolism [14,100].

The rate at which respiration occurs is majorly dependent on the quantity of water uptake by the grains [93,100,101]. Then, the seed modification is further encouraged by the gibberellic acids, a plant hormone that triggers movement from the embryo to the aleurone layer during steeping [101,102]. Additionally, Ref. [103] noted that an increased soaking period may result in anaerobic fermentation of soaked soybeans due to reserved foods limited availability causing the sprout lengths to be decreased. The sprout length differences showed the same trends, suggesting that differences in steeping time in this study were primarily due to water immersion duration. In addition, it showed that the 48 h steeped BGN malt had high water uptake with shorter sprout length due to increased steeping time. This resulted in over-steeping, irregular and depressed germination, resulting in grain death known as induced water sensitivity [1,104,105].

**Figure 1:**
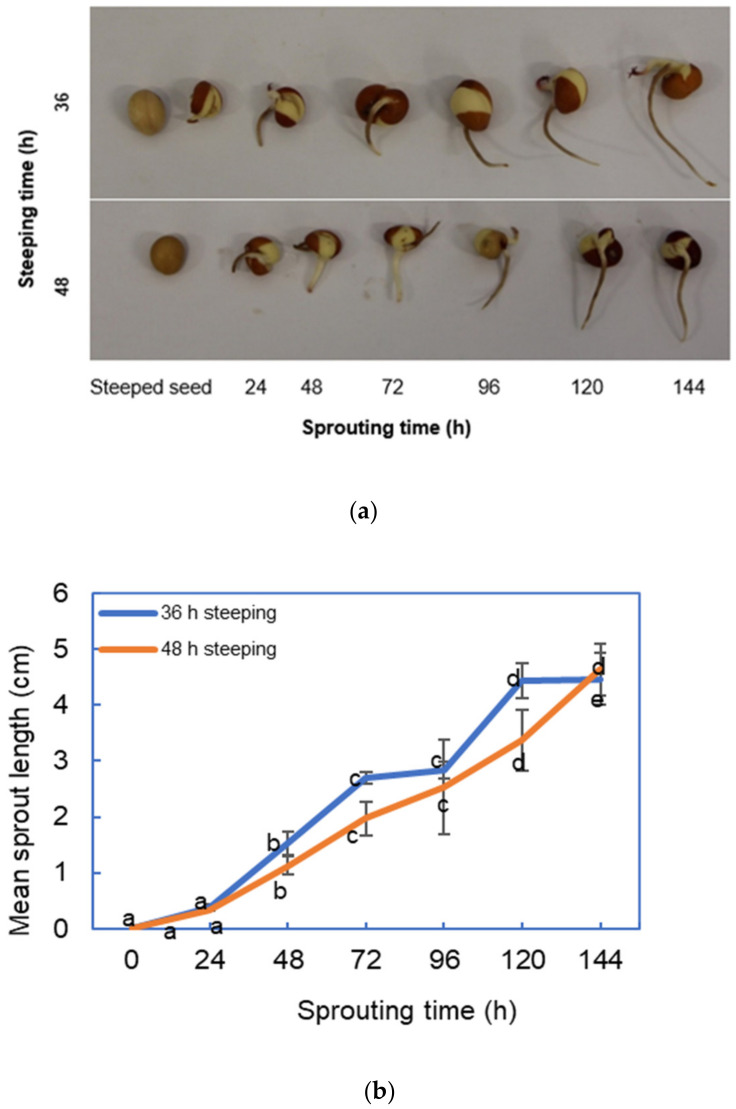
Bambara groundnut (**a**) sprout length changes from 24 to 144 h and (**b**) increase in sprout length from 0 to 144 h sprouting time ^1^. ^1^ Series with different alphabets differ significantly (*p* < 0.005).

The sprouting time from 24 h to 144 h affected the sprout length of the malted BGN seeds. The sprout length increased with the sprouting time, as shown in Figure 1a,b. The sprouted BGN seeds were significantly (*p* = 0.000) different, as illustrated in Figure 1b. The highest sprout length of 4.47 and 4.63 cm were observed for 36 and 48 h steeped BGN malts at 144 h sprouting. However, the 36 h steeped seeds exhibited higher sprout lengths from 24 to 120 h of sprouting, but the sprout length of the 48 h steeped seeds was longer at 144 h. These differences in sprout length indicated that sprout length increased with sprouting time [57,106]. Likewise, Dahiya et al. [107] noted that sprouting time affected the sprout length of grains. Similar observations were made by [108,109] in soybean, BGN, and cowpea seeds, where the sprouting duration affected their length.

The progressive increase in sprout lengths from 24 to 144 h of sprouts for the two steeping regimes could be attributed to the successful modification of the seeds. The sprout length depends on the modification of the grains during sprouting. The gibberellic acid, a plant hormone, diffuses into the aleurone layer to signal the production of enzymes for stimulating acrospires growth [101,110,111]. The 36 h steeped seeds sprouted from 24 to 144 h had the highest sprout lengths. This result is similar to the study by [112], where 24 h steeping time showed higher sprout length than 48 h attributed to prolonged soaking of 48 h, which inhibited sprout length caused by the accumulation of sucrose, which is an inhibitor of α-amylase [113]. Since steeping and availability of oxygen activate α- and β-amylase, which encourages increased acrospire length [112,113], a proper steeping time is necessary for sprouting BGN seeds to give desirable bioactive compounds that can be used to develop functional food products. Since steeping and availability of oxygen activate α- and β-amylase, which encourages increased acrospire length [112], a proper steeping time is necessary for sprouting BGN seeds to give desirable bioactive compounds that can be used to develop functional food products. In this study, steeping for 36 h and sprouting for 96 h would be an optimal condition for producing sprouted BGN to avoid the loss of bioactive components that may be beneficial to health.

### 3.3. Effect of Steeping and Sprouting Time on the Colour of Bambara Groundnut Malt

The CIE L*a*b* colour space coordinates of the steeped BGN seeds as affected by steeping times is in Table 1. The mean lightness (L*), redness (a*), and yellowness (b*) for 36 h steeping were 76.11, 2.93, and 11.45, respectively. The lightness (L*), redness (a*), and yellowness (b*) for 48 h were 75.60, 3.42, and 12.81, respectively. The chroma (C*) and hue angle (h°) were 12.02 and 75.67o, respectively. Based on steeping time, there was a significant (*p* = 0.003) difference in the lightness (L*), where the 36 h steeped BGN malt was lighter than the 48 h steeped seeds. The positive redness (a*) indicated that both steeping times exhibited redness in colour. However, the redness did not show a significant difference (*p* = 0.157) for the 36 and 48 h steeping times. The steeped BGN malts differed significantly (*p* = 0.002) in yellowness (b*) between 36 and 48 h steeping times. The chroma (C*) and hue angle (h°) were not significantly different for the 36 and 48 h steeping times.

The steeping time increased the malts’ redness (a*) and yellowness (b*). The increase in yellowness agrees with the results of [114], where soybean was soaked to measure the soybean’s colour change and the soaking water; the colour change was attributed to the degradation of red pigments into yellow colour. The rise in the malted BGN seeds yellowness could, however, be explained to be due to the leaching of plant pigments (water-soluble colour compounds) such as chlorophyll, xanthophyll, and carotene lost during steeping [115]. The decrease in lightness (L*) and increased redness (a*), respectively, were also due to pigment transfer from grain coat to endosperm and the onset of modification of grains. Likewise, changes in structure, disruption, the disintegration of molecules, and bond breakage decreased lightness by breaking down carbohydrates and proteins [74,116,117].

Although there was no significant difference in the chroma (C*) and hue angle (h°), there was an indication of an increase in chroma (C*) and a decrease in hue angle (h°) at an increase in steeping time. The chroma represents the colour intensity or strength of colour, starting from grey [118]. The increased chroma due to the increase in steeping time suggested that the BGN malts had a less saturated angle [119]. The hue angle (h°) is the quality attribute of colour defined as reddish, greenish, and yellowish for 90, 180, and 270° as perceived by human eyes [118,120]. A higher hue angle represents a lesser yellow character; thus, the hue angle (h°) for both steeping times were between 0° and 90°, where 0° represents the red colour, and 90° represents the yellow colour. Therefore, the hue angle of the BGN malts indicates that the BGN malts colour was reddish-yellow [121]. The differences in steeping time had an impact on the BGN malt colour quality, and this is in agreement with the study of [115], where chickpeas exhibited darker seeds after steeping, attributed to the leaching of water-soluble phenolics consisting of yellow/red compounds such as anthocyanidin and flavanols. However, the leaching period is dependent on the soaking time and temperature [114,115], leading to darker seeds [122].

Sprouting time affected the lightness (L*) of the 36 and 48 h steeped BGN seeds ranging from 68.02 to 82.67 and from 72.77 to 79.97, respectively, as shown in Table 2. The redness and yellowness were significantly (*p* = 0.000) different for the 36 and 48 h steeped BGN malt sprouted from 0 to 144 h. The redness (a*) and the yellowness (b*) for 36 h steeped BGN malts were from 0.76 to 6.22 and from 8.08 to 10.18, respectively (Table 2), while the 48 h steeped BGN malts were from 1.70 to 5.00 and from 8.18 to 13.14, respectively. The increase in sprouting time led to a reduction in lightness, making the malts darker. The malts positive values for redness (a*) and yellowness (b*) indicated that the BGN malts had more red and yellow pigments [123]. There was an increase in the redness (a*) and yellowness (b*) of the BGN malt as sprouting time increased from 24 to 144 h with a significant (*p* = 0.000) difference. Observed changes in the BGN malt colour can be attributed to the melanoidins (colour compounds associated with heat) due to the Maillard reaction during kilning [118].

The Maillard reaction is associated with the interaction of amino acids and saccharides in sprouted grains produces Maillard Reaction Products (MRPs) during kilning due to the temperature and time of drying [124,125,126].

The chroma and hue angle of BGN malts for the 36 h steeping time ranged from 8.26 to 14.47° and from 58.51 to 83.40°, respectively, from 24 to 144 h of sprouting. The chroma and hue angle of BGN malts for the 48 h steeping ranged from 6.55 to 25.78° and from 54.15 to 83.56°, respectively. There was a significant (*p* = 0.000) difference in chroma (C*) from 24 to 144 h of sprouting for the 36 and 48 h steeping times. However, there was no significant (*p* = 0.139) difference in the hue angle (h°) from 24 to 144 h of sprouting. The hue angle is the primary colour characteristic that describes the red, green, blue, and yellow colours the human eye perceives [123,127,128]. It measures an angle of 0° to 360° (0° and 360° = red, 90° = yellow, 180° = green, and 270° = blue) [118,123]. The two steeping times hue angle were affected by sprouting with a range of 58.51° to 83.57° for 36 h steeped BGN malt and 54.15° to 83.56° for 48 h steeped BGN malt. The two steeping regimes’ hue angles for the sprouted BGN malts were less than 90°, indicating reddish-yellow as described in previous studies [120,129].

**Figure 2:**
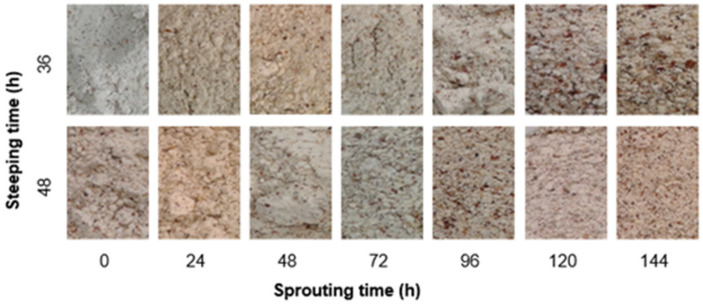
Colour characteristics of sprouted Bambara groundnut.

The changes in lightness, redness, yellowness, hue angle, and chroma of malted BGN in this studies could be attributed to differences in the steeping and sprouting times, producing different soluble sugars and protein content (amino acids) [6,130,131]. The changes in the colour coordinates of the malted BGN correlate with sprouted mung bean flour and malted sorghum-soy becoming darker with the increase in germination time due to the enzymatic hydrolysis during germination [132,133]. Additionally, studies of BGN based on colour has shown that seed coat colour affected seed germination attributed to the impact of the hydrolytic potential of the BGN and Maillard reaction during drying treatment [134,135]. The colour changes for the sprouted BGN seeds are shown in Figure 2.

The colour formation during malt processing significantly impacts the appearance and acceptability of food malt products [119,136]. Lightness (L*), redness (a*), yellowness (b*), chroma, and hue angle are important indications of the quality of the product and market worth [137,138]. Recently, consumers have been asking for natural food colours in food production, which has increased the demand for malted grains as colourants in food production [139,140,141]. The BGN malt colour characteristics, reddish-yellow in this study, are a colour combination that is important in food processing industries due to its ability to grab attention, stimulate tastebuds, and increase appetite [142,143]. Hence, the malts produced from BGN seeds could be a natural source of colour in food production to enhance food colour.

### 3.4. Effect of Steeping and Sprouting Time on the pH of Bambara Groundnut Malt

The mean triplicate measurements of pH for the 36 and 48 h steeped BGN seeds were 6.11 and 6.13, respectively. The 48 h steeped BGN seeds’ pH values were significantly (*p* = 0.001) higher than those of the 36 h steeped seeds. The increase in pH exhibited could be attributed to increasing hydrogen ion content due to the biological activity of the carbohydrates and other food nutrients to produce organic acids [144,145].

**Figure 3:**
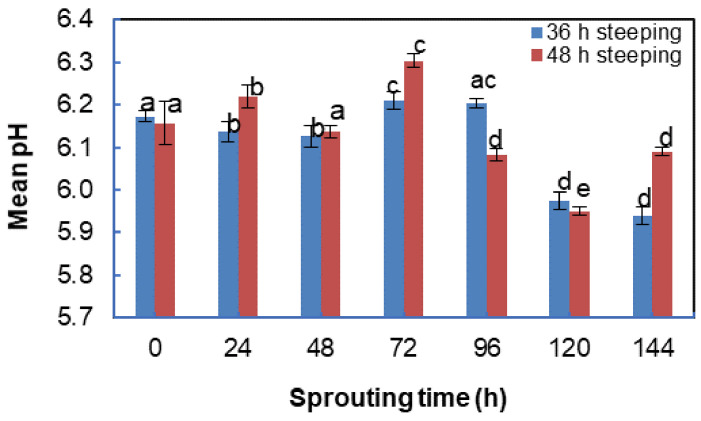
pH changes during sprouting of Bambara groundnut ^1^. ^1^ Bars with different alphabets (a, b, c, d and e) differ significantly (*p* < 0.05).

Sprouting from 24 to 144 h showed a significant (*p* = 0.000) difference for both steeping regimes. The pH for 36 h steeped BGN malt ranged from 5.94 to 6.21, while the 48 h steeped BGN malt values ranged from 5.95 to 6.22, as shown in Figure 3. The decrease in pH with the increase in sprouting times has been attributed to the lipase activity, which acts on triacylglycerols to convert them into free fatty acids required for the generation of energy [81,146]. It was also suggested that the decrease in pH of sprouted finger millet flour might be due to the production of organic acids during the sprouting time [147]. Similar to this work are the studies on germinated maize and horse gram flour, where germination resulted in a reduction in pH [148,149]. Handa et al. [149] also reported that the decrease in pH was attributed to the reduction in enzyme secretion that hydrolyses complex organic molecules such as phytic acid and protein into simpler acidic compounds such as phosphate and amino acids, respectively.

### 3.5. Effect of Steeping and Sprouting Time on the Protein Content of Bambara Groundnut Malt

The 36 h steeped BGN malt mean protein from the triplicate analysis was 19.98%, and the 48 h steeped BGN was 20.55%. Based on the Kruskal–Wallis test, protein distribution is the same across the steeping time (h), thus showing no significant difference (IBM SPSS version 26). The protein distribution with no significant difference was also observed by [150] and [33] where there were no significant differences in the protein contents of amaranth and goat pea (*Securigera securidaca* L.) subjected to varying steeping times. However, [151] and [149] reported that steeping time increases the protein content in mung bean and amaranth grains. The increase is attributed to the change in the starch, water, and lipid components in the grains during steeping, which may have altered the protein’s proportion on dry weight matter [72,152]. The increase in the protein content could also be due to increased free amino acids and peptides and the rise in non-protein nitrogenous contents during steeping [55,109,153]. These contrary results were attributable to different factors, including species and variety, seed availability, and environmental conditions [150].

**Figure 4:**
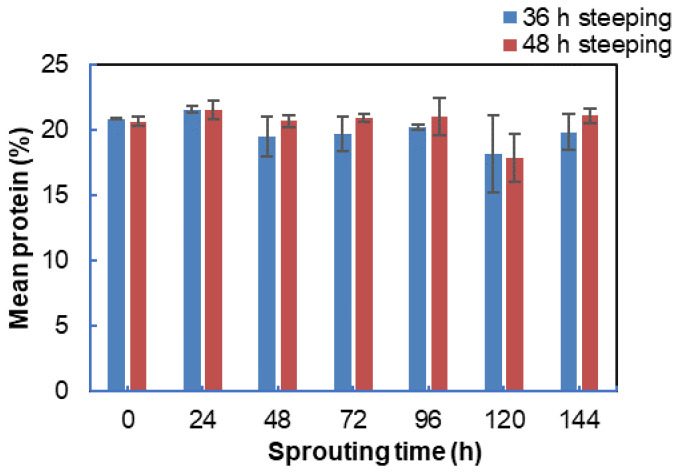
Protein content of sprouted Bambara groundnut from 0 to 144 h.

The crude protein based on the sprouting time showed no significant difference from 0 to 144 as analysed by the Kruskal–Wallis test in Figure 4. The result is similar to the studies on germinated legumes, mung beans, goat pea, and light brown speckled kidney beans [33,37]. Several studies have shown that sprouting increases the protein content of lupin, peas, chickpea, moth beans, soya beans, and mung beans [32,34,35,40,94,154,155,156]. Researchers have also observed lower protein content in sprouted legumes, resulting from seed types and conditions of sprouting [34,35,157,158]. The change in protein content has been attributed to the interaction between protein degradation and biosynthesis as steeping and germination times increase [33,149,159,160]. Additionally, the legumes’ protein content depends on the type of legume seeds and processing conditions such as steeping and sprouting [161,162,163,164].

### 3.6. Effect of Steeping and Sprouting Time on α- and β-Amylase Activities of Bambara Groundnut Malt

Steeping at 36 and 48 h showed a significant (*p* < 0.05) effect on the α- and β-amylase activities of the malted BGN seeds, as shown in Table 3. Steeping at 36 h had mean (triplicate analysis) α-amylase activities of 0.14 CU/g, while β-amylase activities were 0.21 BU/g. While steeping at 48 h had mean α-amylase activities of 0.17 CU/g and β-amylase of 0.22 BU/g. The 48 h steeped BGN malt has higher α- and β-amylase activities than the 36 h steeped BGN malt. The difference is in agreement with the research on the amylase activities of mung bean (*Phaseolus aureus),* cowpea (*Vigna catjang*), lentil (*Lens culinaris*), and chickpea (*Cicer arietinum*) [165]. The increase in amylase activity with an increase in steeping time is due to enzymes’ activation during steeping and the penetration of the gibberellic acid by diffusion to the aleurone layers to prompt enzyme synthesis [79,101,102,166]. Based on steeping time, BGN seeds steeped at 48 could, however, give optimal α- and β-amylase activities.

**Figure 5:**
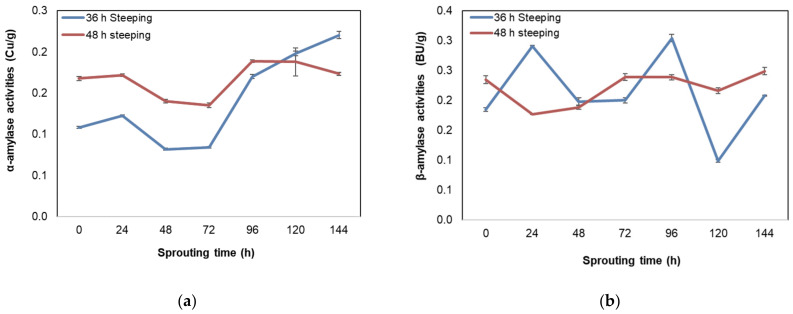
Effect of sprouting time on (**a**) α-amylase activities and (**b**) β-amylase activities of Bambara groundnut.

There was a significant (*p* = 0.000) difference based on sprouting time for the α- and β-amylase activities of BGN malt. The mean α-amylase activities for the 36 steeped malted BGN ranged from 0.11 to 0.22 Cu/g, while the 48 h steeped seeds ranged from 0.17 to 0.19 Cu/g shown in Figure 5a. The β-amylase activities ranged from 0.18 to 0.30 Bu/g and from 0.18 to 0.25 BU/g for the 36 and 48 h steeped seeds, respectively, in Figure 5b. There was an increase in the activities of the α- and β-amylases as sprouting time increased. However, a decrease was observed after 96 h for the β-amylase activities and, thereafter, an increase at 144 h. The increase in the α- and β-amylase activities in BGN malted seeds agreed with the results of enzyme activities of germinated mung bean (*Phaseolus aureus*), cowpea (*Vigna catjang*), lentil (*Lens culinaris*), chickpea (*Cicer arietinum*), and adzuki bean (*Vigna angularis*) increasing with an increase in sprouting time [24,38,167].

The increase in amylase activity resulted from the seeds absorbing water while steeping, subsequently mobilising their dormant reserve [166,168]. The absorbed water then stimulates the embryo to produce gibberellic acid, which influences seed growth and developmental processes, including dormancy and germination [169]. The gibberellic acid then diffuses to the aleurone layer and starts a flow resulting in the synthesis of α- and β-amylase [12,165,169,170]. The increase in β-amylase after the decline at 120 h sprouting time could be due to β-amylase being heat liable [171]. However, based on sprouting times, the 36 h steeping and 96 h sprouting times could be regarded as an optimum to produce an amylase-rich malted BGN seed.

### 3.7. Effect of Steeping and Sprouting Time on Total Polyphenol Activities of Bambara Groundnut Malt

There was a total polyphenols concentration of 0.92 mg and 0.99 GAE/g for the 36 h and 48 h steeping time, respectively. There was an increase in total polyphenols for the 36 h steeped BGN seeds and the 48 h steeped seeds. The increase in total polyphenols for the two steeping regimes showed that polyphenols content improved with an increase in steeping times of BGN seeds. The increase in total polyphenol activities is in agreement with the study of beans and pinto, where total polyphenolic compounds increase with soaking times [172].

The increase is attributed to polyphenols solubilisation due to water uptake during steeping [51,173,174]. However, most studies have shown that total polyphenols are reduced when legumes are steeped, depending on the soaking conditions (time and temperatures) and legume varieties [87,96,175,176,177,178]. Barimalaa and Anoghalu [179], in their study, however, noted that cold-soaking overnight had a minimal effect on the rate of polyphenols loss in BGN seeds.

**Figure 6:**
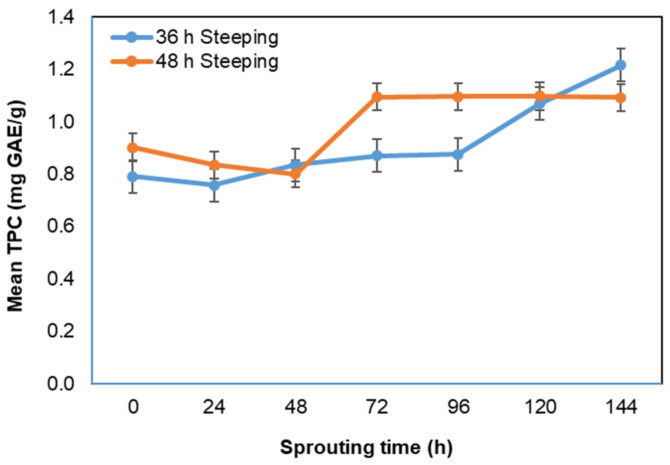
Total polyphenolic contents (mg GAE/g) with sprouting time.

Sprouting time had a significant difference (*p* = 0.000) on the total polyphenol content of sprouted BGN seeds, as shown in Figure 6. The total polyphenol increased with sprouting time, with the highest concentration at 144 h (1.22 mgGAE/g) sprouting for the 36 h steeping while 48 h steeping was at the 120 h (1.10 mgGAE/g) sprouting. The increase in total polyphenolic concentration indicated an improvement in total polyphenols based on sprouting time [180].

Total polyphenol contents increased significantly (*p* = 0.000) with an increase in steeping and sprouting time. The relative increase in total polyphenol contents during sprouting was reported by [17,181] for mung beans, black beans, and soybeans; by [182] and [117] for faba beans, chickpea seeds, lentils seeds, fenugreek seeds; and by [183] for lupine seeds. An increase in phenolic compounds was observed in soybean and mung beans with an increase in germination time [180,184]. The increase might be attributed to the formation of phenol compounds during sprouting time. The increase in polyphenols could also be due to condensed tannin’s solubilisation during seed soaking [117,185,186]. Furthermore, the increase in polyphenols has been attributed to the link between enzyme activity and water availability during malting [40]. The absorption of water activates the dormant enzymes to stimulate growth. The stored enzymes are hydrolysed, making the enzyme-substrate produce new products (phenolic). The bound phenolic compounds become free by activating endogenous enzymes during germination [26,187]. Similar findings were reported by [187] in germinated chickpea flour, by [188] in pea and black bean, by [189] in lentil sprouts, and by [26] in four legumes. However, the increase in total polyphenols as sprouting time increases was attributed to the phenolic composition changes caused by the endogenous enzyme activation and seeds’ biochemical metabolism during the sprouting process [190,191]. The increase in total polyphenolic contents in this study during steeping and sprouting times showed that BGN malt is an antioxidant-rich product and was improved with steeping and sprouting time, and this could, however, be beneficial for consumers with oxidative stress-associated diseases [191,192]. These findings indicated that an increase in total polyphenols of sprouted BGN has the potential for use in the nutraceutical industry to offer some health benefits to consumers.

### 3.8. Effect of Steeping and Sprouting Time on the Antioxidant Activities of Bambara Groundnut Malt

**Figure 7:**
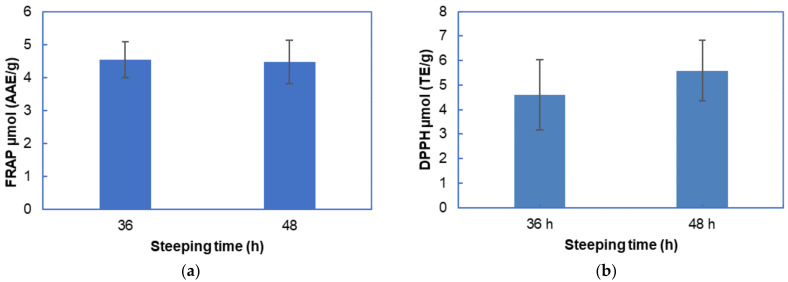
Effect of steeping time on the antioxidant activities of Bambara groundnut: (**a**) FRAP assay and (**b**) DPPH assay.

Steeping BGN seeds for 36 h had a mean antioxidant activity of 4.55 µmol (AAE/g) for FRAP and 4.59 µmol (TE/g) for DPPH assays, respectively. For both assays, the 48 h steeping antioxidant activities was higher than the 36 h BGN steeped seeds. There was a significant (*p* = 0.000) difference in the FRAP and DPPH antioxidant concentration in the 36 and 48 steeping times, as shown in Figure 7. The changes in the FRAP and DPPH assayed antioxidant concentrations are attributed to leakage of antioxidant compounds in soaking water [117,193]. Additionally, soaking for longer times resulted in higher biochemical metabolism of the seeds by releasing more phenolic compounds, resulting in increased antioxidant activity, as shown in 48 h steeped BGN seeds [194]. Additionally, the authors of [195] hypothesised that soaking water remaining from the seed may have extracted the soluble free and linked phenolic, thus increasing the antioxidant capacity.

Sprouting from 24 to 144 h for both 36 and 48 h steeped BGN malt FRAP antioxidants did not show a significant difference, as shown in Table 4. However, the 48 h steeped seeds sprouted for 120 h had the highest antioxidant activity. There was a significant (*p* = 0.005) increase from 24 to 144 h in sprouting in the DPPH radical scavenging antioxidant concentrations for the 36 and 48 h steeped BGN malt difference.

The increase in antioxidants could be due to the activation of the natural endogenous antioxidants that occur in legumes during sprouting [26,189]. However, the increase in and modification of antioxidants in legumes depends on grain types and malting conditions [189]. Research on the malting process of legumes such as soybeans, pea, mung beans, lentils, cowpea, jack bean, dolichol, and mucuna showed that an increase in sprouting time increased antioxidant activities [173,181,188,190,196].

The result of the antioxidant activities using FRAP and DPPH assays in this study indicated that steeping and sprouting times increased antioxidant activities in BGN seeds. However, the DPPH free radical scavenging antioxidant activities were higher, suggesting that they contain components that can scavenge free radicals to increase antioxidant activities [192]. Increased antioxidant activity in BGN malt was due to the release of phenolic compounds bound to the cell structure during steeping and sprouting times [197]. Steeped BGN seeds at 48 h, sprouting for 120 h and assayed using the DPPH, gave antioxidant-rich BGN malt. The higher antioxidants in the 48 h steeped and 120 h sprouted seeds presented the potential of BGN as a legume with incredible beneficial properties for food and industrial applications.

## 4. Conclusions

The steeping and sprouting times affected the physicochemical characteristics of BGN seeds. The colour of BGN was reddish-yellow, which is a desirable colour combination for improving or enhancing the colour of food produced. The malting process significantly affected the amylase enzyme activity of BGN seeds. The steeping and sprouting processes increased the amylase activities of the BGN malt for both 36 and 48 h steeping times. However, an amylase-rich BGN malt could be produced by steeping for 36 h and sprouting for 96 h. In addition, total polyphenols and antioxidants improved during the steeping and sprouting processes. Steeped BGN seeds for 48 h, sprouted for 120 h, resulted in an antioxidant-rich BGN malt that could have nutraceuticals benefits. Bambara groundnut malt under properly controlled malting conditions could be perfect for new food production, such as barley malt. Malt products are a good source of colour, amylase, and antioxidants, and food industries rely on them for food production. Furthermore, BGN is a climate change crop that could ease the demand for malt uses and add beneficial properties to food and industrial applications.

## Figures and Tables

**Table 1:** Steeped BGN seeds colour characteristics ^1^.

	Steeping Time (h)	
Colour Parameters	36	48
Lightness (L*)	76.11 ± 6.02 ^a^	75.60 ± 4.09 ^b^
Redness (a*)	2.93 ± 2.27 ^a^	3.42 ± 1.27 ^a^
Yellowness (b*)	11.45 ± 2.05 ^a^	12.81 ± 2.57 ^b^
Chroma (C*)	12.02 ± 2.08 ^a^	13.71 ± 10.40 ^a^
Hue Angle (h°)	75.67 ± 10.40 ^a^	71.10 ± 15.58 ^a^

^1^ Mean values ± standard deviation of triplicate determinations, mean values in the same row with different letters (a and b) are significantly (*p* ≤ 0.05) different. L*: Lightness; a*: Redness, b*: Yellowness, C*: Chroma, h^o^: Hue Angle.

**Table 2:** The effect of sprouting time on the colour of Bambara groundnut malts ^1^.

36 h Steeping					
Sprouting Time (h)	L*	a*	b*	Chroma	Hue Angle (h°)
0	82.67 ± 0.43 ^a^	0.76 ± 1.08 ^a^	8.08 ± 0.88 ^a^	8.16 ± 0.82 ^a^	82.11 ± 4.32 ^a^
24	81.59 ± 1.00 ^b^	2.40 ± 1.63 ^a^	10.05 ± 0.33 ^b^	10.42 ± 0.34 ^b^	76.64 ± 9.01 ^ab^
48	79.56 ± 0.08 ^c^	1.63 ± 1.15 ^a^	12.46 ± 0.86 ^c^	12.60 ± 0.87 ^cd^	82.53 ± 5.09 ^a^
72	79.15 ± 0.07 ^c^	1.45 ± 0.32 ^a^	12.83 ± 0.51 ^c^	12.91 ± 0.53 ^cd^	83.57 ± 1.26 ^a^
96	74.50 ± 0.54 ^d^	2.49 ± 1.40 ^a^	13.21 ± 1.13 ^c^	13.50 ± 0.80 ^ef^	79.06 ± 6.97 ^ab^
120	67.27 ± 0.31 ^e^	5.58 ± 0.71 ^b^	13.33 ± 0.70 ^c^	14.47 ± 0.58 ^f^	67.27 ± 3.23 ^bc^
144	68.02 ± 0.17 ^e^	6.22 ± 1.83 ^b^	10.18 ± 1.25 ^b^	12.06 ± 0.56 ^c^	58.51 ± 10.16 ^c^
**48 h Steeping**					
0	79.97 ± 0.05 ^a^	3.56 ± 0.31 ^abc^	8.18 ± 1.40 ^a^	13.20 ± 2.22 ^abc^	66.21 ± 3.65 ^a^
24	80.14 ± 1.12 ^a^	2.43 ± 0.52 ^cd^	11.57 ± 1.04 ^b^	6.55 ± 2.64 ^ab^	54.15 ± 39.56 ^a^
48	78.17 ± 0.26 ^b^	1.70 ± 0.97 ^d^	14.83 ± 0.70 ^c^	3.96 ± 3.45 ^a^	83.56 ± 3.40 ^a^
72	74.43 ± 0.43 ^d^	4.23 ± 0.68 ^ab^	13.34 ± 0.44 ^bc^	18.67 ± 6.02 ^cd^	72.48 ± 2.12 ^a^
96	75.49 ± 0.31 ^c^	3.79 ± 1.55 ^abc^	14.56 ± 2.70 ^bc^	16.48 ± 11.27 ^bcd^	74.78 ± 7.19 ^a^
120	68.22 ± 0.74 ^f^	5.00 ± 0.65 ^a^	14.02 ± 1.14 ^bc^	25.78 ± 6.75 ^d^	70.23 ± 3.92 ^a^
144	72.77 ± 0.07 ^e^	3.22 ± 0.84 ^bcd^	13.14 ± 2.38 ^bc^	11.34 ± 5.41 ^abc^	76.31 ± 1.57 ^a^

^1^ Mean values ± standard deviation of triplicate determinations, mean values in the same column within steeping time with different letters (a, b, c, d, e and f) significantly differ (*p* ≤ 0.05), L*: Lightness; a*: Redness, b*: Yellowness.

**Table 3:** α- and β-amylase activities as affected by steeping time ^1^.

Amylase Activities	Steeping Time (h)	
	36	48
α-amylase (CU/g)	0.14 ± 0.05 ^a^	0.17 ± 0.02 ^b^
β-amylase (BU/g)	0.21 ± 0.07 ^a^	0.22 ± 0.03 ^b^

^1^ Mean values ± standard deviation of triplicate determinations. Mean in the same row followed by different letters (a and b) are significantly (*p* < 0.05) different.

**Table 4:** Sprouted BGN seeds antioxidant activities ^1^.

Antioxidant Assay	Sprouting Time (h)	Steeping Time (h) ^1^	
		36	48
FRAP umol (AAE/g)	0	5.14 ± 0.38 ^a^	3.90 ± 0.17 ^a^
	24	5.12 ± 0.38 ^a^	3.80 ± 0.16 ^a^
	48	4.53 ± 0.06 ^b^	3.80 ± 0.19 ^a^
	72	4.47 ± 0.24 ^b^	4.93 ± 0.48 ^bc^
	96	3.60 ± 0.02 ^c^	4.60 ± 0.17 ^b^
	120	4.30 ± 0.10 ^b^	5.21 ± 0.09 ^c^
	144	4.72 ± 0.13 ^ab^	5.14 ± 0.55 ^bc^
DPPH umol (TE/g)	0	3.68 ± 1.11 ^a^	4.57 ± 0.99 ^a^
	24	3.52 ± 0.80 ^a^	4.47 ± 0.80 ^a^
	48	3.75 ± 1.52 ^a^	5.04 ± 0.21 ^a^
	72	4.38 ± 1.66^ab^	5.44 ± 1.64 ^a^
	96	4.94 ± 0.98 ^ab^	6.11 ± 1.06 ^a^
	120	6.25 ± 0.12 ^b^	7.44 ± 0.41 ^b^
	144	5.62 ± 1.59 ^ab^	6.08 ± 0.19 ^ab^

^1^ Values are mean ± standard deviation of triplicate values; mean values in the same column followed by different letters (a, b and c,) are significantly (*p* < 0.05) different FRAP and DPPH activities.

## Data Availability

No additional data were generated other than the ones reported in the manuscript.
